# Choosing the Probe for Single-Molecule Fluorescence Microscopy

**DOI:** 10.3390/ijms232314949

**Published:** 2022-11-29

**Authors:** Chiara Schirripa Spagnolo, Stefano Luin

**Affiliations:** 1 National Enterprise for Nanoscience and Nanotechnology (NEST) Laboratory, Scuola Normale Superiore, Piazza San Silvestro 12, I-56127 Pisa, Italy; 2 National Enterprise for Nanoscience and Nanotechnology (NEST) Laboratory, Istituto Nanoscienze, Consiglio Nazionale delle Ricerche (CNR), Piazza San Silvestro 12, I-56127 Pisa, Italy

**Keywords:** fluorophores, fluorescent probe, fluorescent labeling, single-molecule imaging, fluorescent microscopy

## Abstract

Probe choice in single-molecule microscopy requires deeper evaluations than those adopted for less sensitive fluorescence microscopy studies. Indeed, fluorophore characteristics can alter or hide subtle phenomena observable at the single-molecule level, wasting the potential of the sophisticated instrumentation and algorithms developed for advanced single-molecule applications. There are different reasons for this, linked, e.g., to fluorophore aspecific interactions, brightness, photostability, blinking, and emission and excitation spectra. In particular, these spectra and the excitation source are interdependent, and the latter affects the autofluorescence of sample substrate, medium, and/or biological specimen. Here, we review these and other critical points for fluorophore selection in single-molecule microscopy. We also describe the possible kinds of fluorophores and the microscopy techniques based on single-molecule fluorescence. We explain the importance and impact of the various issues in fluorophore choice, and discuss how this can become more effective and decisive for increasingly demanding experiments in single- and multiple-color applications.

## 1. Introduction

Fluorescence microscopy has become an outstanding technique for observing biological samples thanks to its exquisite selectivity and specificity [1,2]. The continuous evolution in this field allows investigations at the single-molecule level. Their main advantage is the exploration of intermediate states and heterogeneities in biomolecule behavior, since they allow observation of phenomena obscured in conventional “average”/“bulk” approaches [3,4,5]. This has been exploited in studying many biological topics, e.g., transcription [6,7], cell membrane structure [8,9], signaling [10,11], molecular interactions [12], protein folding [13,14], diffusion [15], and endocytic and exocytic pathways [16].

Advanced single-molecule applications require synergic development in multiple fields [17,18], from the evolution of fluorescence probe design and bioconjugation chemistry [19] to the refinement of microscopy techniques and progress in experimental equipment, such as detectors [20,21], to new computational approaches [22,23].

The choice of the fluorescent probe can dramatically influence the performance of single-molecule experiments by affecting the achievable spatiotemporal resolution and range and, ultimately, the accessible information. The full potential of a modern complex microscopy setup could not be fully exploited due to limitations imposed by the used probe. At the same time, the precision and accuracy of the analysis algorithms are greatly reduced by inadequate performance of the selected fluorescent probe [24].

Moreover, the effects of different properties of the fluorophores on the process under investigation have to be considered, since they could alter results in different applications, e.g., by altering the function or in general the behavior of the labeled molecule.

The variety of available fluorescent labels is now very wide, so it is essential to follow some criteria for the optimal choice. This is always the case in fluorescent microscopy [1], but these criteria must become more rigorous, in order to extract the valuable insights of a single-molecule experiment. Here, we aim to provide a comprehensive review covering the key points in the choice of a probe for single-molecule fluorescence microscopy. We start by exposing some examples of how this has been performed in the past (Section 2, and more is reported in the following sections); then we discuss some general aspects to be considered, i.e., signal-to-noise ratio (SNR; Section 3) and aspecific interactions of the probes (Section 4). Section 5 contains a description of the three classes of fluorophores (fluorescent proteins, Section 5.2; organic dyes, Section 5.3; fluorescent nanoparticles, Section 5.4), and a discussion of their advantages and drawbacks in single-molecule fluorescence-based techniques, presented in Section 5.1.

## 2. Comparisons of Fluorophores for Single-Molecule Applications in the Literature

Identification of the best dyes in specific single-molecule applications is often based on comparisons of one or a few of the probe features (Table 1).

For example, Vandenberk et al. compared photostability, brightness, fluorescence lifetime and Förster resonance energy transfer (FRET) performance in single-molecule FRET (smFRET, see Section 5.1) on freely diffusing double-stranded DNA molecules [25]. Considering green (Atto488 and Alexa488, called “blue” in the paper) and far-red (Atto647N, Alexa647, StarRed, and Atto655) dyes, Alexa488 and Atto647N were overall the best. The authors specified that dye performance might differ in experiments on immobilized molecules. Another study analyzed aspecific interactions of dyes with different kinds of lipids and found that Atto 647N interacted strongly with all considered lipids [26]. In general, the authors recommended blue-excitation fluorophores such as Alexa 488 or Atto 488 for their few or no interactions with different kinds of lipids; for smFRET, they selected the blue–red pair Alexa 488 and Alexa 647 and the green–red pair Atto 532 and Alexa 647 [26]. Various fluorophores were compared also for single-molecule tracking exploiting ACP-derived tags [28] or SNAP-tag [29] fused proteins. The first study selected Abberior STAR 635p as the best candidate in labeling the tropomyosin receptor kinase A (TrkA) receptor, considering aspecific interactions, mean fluorescence intensity and photostability; however, the authors stated that this choice is not universal for labeling receptors, and indeed they obtained different results in dye comparisons on a different receptor [28]. The second work selected Dy 549 and CF 640 as the best choices to label the epidermal growth factor receptor (EGFR) considering aspecific interactions and photostability. In this work, the authors anticipated that their results were probably not directly translatable to different chemical tags, such as HaloTag or ACP-based tags.

Discrimination amongst different fluorophores relied also on considering the impact of labeling on the function or general behavior of the labeled molecules. For example, Kügel et al. observed that the rates and equilibrium constant of DNA hairpin opening and closing depended on the characteristics of the organic dyes used to label the hairpin. No stable hairpin was formed with some labeling dyes, possibly because of a steric hindrance in hairpin formation or a repulsive interaction of the dye molecules; conversely, other dyes stabilized the closed-loop conformation [34]. Marchetti et al. reported slower diffusivities for the p75 neurotrophin receptor (p75^NTR^) on the membrane of living cells when labeled with the quantum dot Qdot 655 than with the organic dye Abberior Star 635p. (see Section 5.4) [35]. Another study revealed that the measured diffusion coefficient of the epidermal growth factor receptor (EGFR) in live cells had different values using different organic fluorophores for labeling. The effect was due to varying levels of aspecific interactions with glass substrates and cells that caused the accumulation of immobile spurious spots (see also Section 4) [27]. A recent work demonstrated that it is possible to alter or even inhibit the formation of DNA nanostructures using different organic dye combinations for labeling the DNA strands [36]. Hayashi-Takanaka et al. conducted a systematic investigation on most of the green, red, and far-red commercial organic dyes available in 2014. They were conjugated with antigen-binding Fab fragments directed against specific histone modifications. Differences in the degree of correct intracellular localization were observed, partly explained by an altered affinity of Fab fragments after dye conjugation [37].

The examples reported in this section confirm that there is no universal best choice of a fluorophore for single-molecule microscopy. In the following, we analyze all the aspects to consider in choosing fluorophores under the conditions of the single-molecule experiment of interest.

## 3. Single-Molecule Signal-to-Noise Ratio

In single-molecule fluorescence microscopy, the first essential requirement is the capability of detecting signals from individual molecules typically labeled with individual probes [38]. The achievement of this goal depends on the SNR parameter, defined as the ratio between the intensity of the signal of interest (above background intensity) and the associated noise.

In order to maximize dye signal intensity, usually a first evaluation relies on the selection of dyes with the highest brightness. This is the product of the absorption extinction coefficient at the excitation wavelength and the quantum yield for the dye emission. Tables of their values are available in the literature or from commercial suppliers, but these values are usually reported only for the maximum absorption wavelength; they must be considered alongside the complete absorption spectrum and the effective excitation wavelengths available in the experimental setup. Equally important for a careful dye comparison is the evaluation of collection efficiency, which depends on the dye emission spectra and the experimental detection filters. Some online tools allow visualizing excitation sources, dyes and filters spectra for more realistic estimations [39,40,41,42,43,44,45,46]. Of note, the tools differ in the available dyes and not all of them include some dyes useful for single-molecule studies. FBbase is a fluorescent protein database, but provides a Spectra Viewer [45] that allows selecting other fluorescent probes, such as organic dyes or Qdots as well. Its list of dyes is very comprehensive, including some optimal candidates for single-molecule applications [30,47,48] not reported by other viewers. An interesting feature, not available in other online tools, is the possibility to select the detector (besides dyes, filters and excitation sources) from a list of models of different brands or by inserting a custom one, in order to visualize its quantum efficiency spectrum. This parameter measures the device effectiveness in converting incident photons into electrons, i.e., its detection sensitivity. For single-molecule studies, where very low signals need to be detected, this is another important factor.

**Figure 1:**
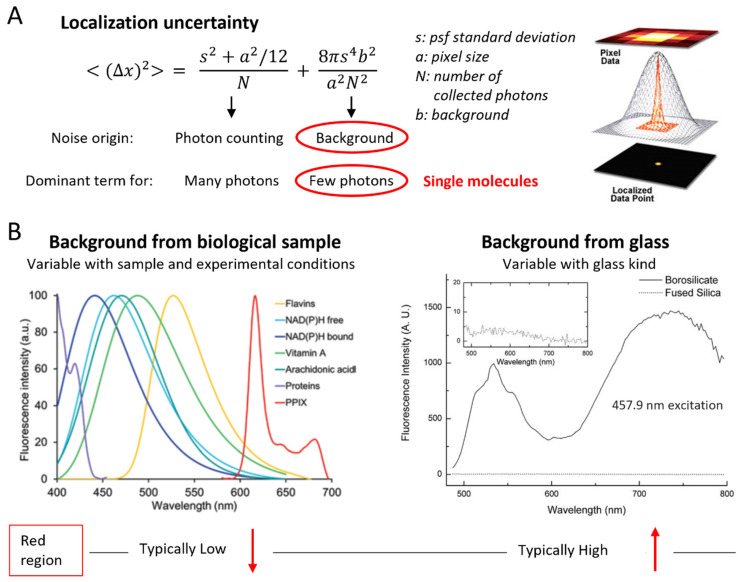
Background impact on localization accuracy affects fluorophore choice for single-molecule applications. (**A**) Expression for the localization uncertainty of stationary fluorophores on a surface as derived in [24]. In conditions of few collected photons, as in the case of single-molecule imaging, the dominant contribution comes from the background. The image on the right illustrates a possible single-molecule localization procedure: raw pixel data are fitted with a two-dimensional Gaussian function that allows the localization in a subdiffraction-limited spot (reprinted from [71] © 2013 IOP Publishing. Reproduced with permission. All rights reserved). (**B**) Typical spectra from two important background sources. Left: possible contributions from biological samples (reprinted by permission from Springer Nature: Springer, *Methods in Molecular Biology* [72] © 2017 Springer Science + Business Media LLC; PPIX: Protoporphyrin IX); right: optical glass (reprinted by permission from Springer Nature, Springer, Microscopy Techniques. *Advances in Biochemical Engineering* [73], Copyright 2005). The emission at longer wavelengths (red region) tends to be relatively low for the first contribution, but relatively high, especially when excited in the UV to the green region, for the second contribution.

Noteworthily, the different excitation and detection bands of different dyes are associated with different background effects, and these influence the noise part of the SNR and therefore the detection and localization precision of single-point emitters [24] (Figure 1A). Indeed, the two main sources of noise are the shot noise of photons from the dye and the noise in the background created by other sources. Background can arise from dark current, light leakage, scattering and autofluorescence from various elements in the setup, and all these contributions are affected at least by Schottky and readout noises. In low-photon conditions, such as for the localization of single molecules, the noise affecting localization precision is usually dominated by the background [24] (Figure 1A). Localization algorithms for single-molecule applications typically perform a first step for spot detection based on a probabilistic comparison of pixel intensities with background noise [23,49,50,51]. For these reasons, the standard deviation of the background intensity is usually used as noise value in estimating the SNR of a single molecule [49,51].

Of course, noise decreases by using ultrasensitive and low-noise detectors, such as cooled EMCCD (electron-multiplying charge-coupled device) cameras. Nevertheless, characterizing the spectra of different scattering and autofluorescence sources can help to choose the optimal fluorescent probes considering the background corresponding to the excitation and detection bands. Among these sources, the most mentioned in the literature concerning biological applications is the biological sample itself (cells or tissue) [52,53,54,55,56] (Figure 1B). It has been reported that these contributions in the visible range typically tend to decrease with increasing excitation wavelength; therefore, especially for single-molecule studies, red-emitting fluorophores are preferred [57,58]. However, different kinds of samples, such as different cell lines or tissue types, have different autofluorescence spectra and intensities. For example, such differences were observed between normal and malignant human breast cell lines [59], between cell strains (e.g., stabilized and transformed lines of fibroblasts [60]) or between immortalized and carcinogen-transformed human bronchial epithelial cells [61]. Autofluorescence has even become a property exploited as a diagnostic tool, e.g., for diagnosis and monitoring of retinal conditions [62] or for identifying cancer stem cells [63] and cancer in several tissue types [64,65,66,67]. Indeed, the autofluorescence imaging methodology exploits this kind of intrinsic emission from biological samples to characterize states such as metabolism, cell density and proliferation rate, differences between cell types or cellular microenvironment [68,69,70]. Because of the variability of the phenomenon, a characterization of the autofluorescence of the specific sample of interest may be beneficial for more careful optimization of dye choice.

Emission from the biological sample is not the only contribution to the background: other sources are cell medium, fixatives, immersion oil, optical elements or coverslip [74,75,76,77]. These additional elements are often overlooked and rarely mentioned, and it is very difficult to find information on their excitation and emission spectra. Special coverslips made of quartz or fused silica have much lower autofluorescence than standard coverslips made of borosilicate glass [75,78,79,80]. However, they cannot be used in many applications because their refractive index, lower than borosilicate glass, induces aberrations, especially with oil immersion objectives [79]. Moreover, coverslips made of these materials are much more expensive than standard glass coverslips. An exhaustive characterization of absorption and emission spectra of typical coverslips autofluorescence could therefore be useful, but we could not find a satisfying one.

Some works mentioned that standard coverslips made of borosilicate glass generate fluorescence in the red region under blue excitation [73,81], green excitation [73,75,82] and mercury arc lamp excitation [83]. This red emission from glass hinders the use of the spectral region usually preferred based on autofluorescence from biological specimens (Figure 1B). The impact of the two autofluorescence sources should be evaluated thoroughly in the experiment of interest. For example, a popular microscopy technique for single-molecule studies is TIRF (total internal reflection fluorescence) microscopy. It allows observation of processes on cell membranes with excellent resolution and SNR. Its peculiarity is an excitation restricted to a thin layer of about 100 nm near the glass–water interface, containing the bottom membrane of cells adherent to the coverslip. Since cellular autofluorescence mainly originates in deeper regions within the cytoplasm [52,84], a bigger contribution to autofluorescence in this technique likely comes from the coverslip. This is especially true in multichannel studies when visualizing at least two dyes emitting in distinct channels, because an additional and possibly higher autofluorescence in the red channel arises from the blue or green excitations used for the shorter-wavelength dye (Figure 1B). In fact, in [73], multichannel single-molecule TIRF implementation including a red-emitting dye was not possible using standard coverslips because of its autofluorescence background. In this work, single Cy3 molecules could be imaged but single Cy5 molecules could not under simultaneous excitation, owing to the background emitted from the glass in the red region [73]. Exploration and comparisons with a configuration that excludes red-emitting dyes have not yet been reported. It could be based on dyes emitting at shorter wavelengths to minimize coverslip background, since autofluorescence from cells in those bands is already minimized in TIRF.

## 4. Aspecific Interactions

Some kinds of fluorescent probes (see also Section 5) suffer from surface adsorption effects on glasses or other substrates in contact with the aqueous medium containing the biological sample [27,85]. This is a generally observed behavior for proteins and other molecules at interfaces [86], which becomes a challenge in various applications, such as biosensing [87,88] or single-molecule microscopy [27,85].

For example, it has been observed that in single-molecule tracking in live cells, aspecific adsorption significantly affects diffusivity measurements because of immobile spots adherent to the coverslip surface [27,85]. The problem cannot be solved by simply discarding the immobile spots because the molecules of interest can also be slowed down or immobilized, e.g., mobility changes and arrests of membrane receptors are pivotal events for their function [10,89,90]. At the same time, the presence of additional aspecifically adsorbed fluorophores causes an increase in the total density of spots, which hinders the detection and localization of molecules at relatively high densities [49], necessary for analyzing such phenomena as interactions at high dissociation constant.

In cell-free experiments, in which fluorescently labeled molecules are specifically bound to a surface, it is important to include data only from those specifically immobilized and not from the aspecifically adsorbed ones. Moreover, uncontrolled binding can result in molecule denaturation and unreliable results [85,91].

Several kinds of surface modifications have been developed for reducing aspecific adsorption, e.g., by using protein blockers (bovine serum albumin (BSA) or other milk proteins), non-amphoteric hydrophilic polymers (polyethylene glycol and derivatives), zwitterionic polymers (phosphorylcholine, sulfobetaine, carboxybetaine), or mixed materials [88,92]. However, multiple factors complicate their applicability. In some cases, they can be unsuitable for live cell experiments since the passivated surface hinders cell attachment [93], or the procedure includes toxic or incompatible reagents, such as the detergent Tween-20 [94,95]. Moreover, cells tend to further modify the surface (e.g., by removing part of the coating or excreting a sort of extracellular matrix), foiling some methods useful in other situations [85]. For example, many protocols of surface passivation are based on the use of polyethylene glycol (PEG). However, its performance is more effective when combined with Tween-20 [94,95]. Moreover, cells cannot adhere to pure PEG: for experiments with cells, PEG layers must be further modified by adding adhesion peptides or other molecules [96]. Finally, in some cases, it has been observed that PEG coating interferes with the process of interest and needs again to be combined with other treatments [97]. This has led to complicated procedures for sample preparation. The additional molecules may depend on the kinds of cultured cells or the specific applications, even for those without cells [98,99,100], with consequent very laborious optimizations possibly using mixtures of several additional molecules [85,101]. Another important factor for single-molecule fluorescent microscopy is that some surface treatments can increase the background signal [85,97].

Aspecific adsorption is actually not limited to the coverslip surface. Fluorophores also showed spurious interactions with lipid bilayers, challenging, for example, single-molecule studies in supported lipid bilayers or on cell membranes [26,102]. This means that surface passivation might not be sufficient to prevent the phenomenon.

For all the described reasons, it is clear that minimizing aspecific interactions is another essential step in choosing the fluorescent probe for single-molecule microscopy. Some studies have reported comparisons of aspecific interactions for different dyes, in particular for single-molecule applications [26,27,28,29,85,102] (Table 2). The first consideration is that all of them observed high variability in the behavior of the different dyes and identified several ones to exclude since they could impair or falsify single-molecule measurements. Each study analyzed the performance of a distinct subset of available dyes; however, especially considering the ones present in different papers, it is possible to extract interesting observations. A couple of dyes have been identified as the best choice for their low aspecific interactions in separate studies and thus in different conditions. These are Atto 488 and Alexa 488. They resulted in negligible or very low aspecific interactions in: (i) [28], where they were conjugated with coenzyme A and used in labeling with a PPTase system in living cells; (ii) [102], where the authors analyzed their interactions with model lipid bilayers, i.e., with unilamellar lipid vesicles using dialysis; (iii) [26], where the authors investigated their interactions with lipid vesicles (by quantifying the partition into the lipid membrane via fluorescence correlation spectroscopy) and with supported lipid bilayers (by visualizing adsorbed fluorophores via TIRF imaging), considering diverse kinds of lipid with variable charges, head groups and degrees of chain saturation. Alexa 488 showed similar results of negligible aspecific interactions when used to label anti-EGFR affibody in live cells cultured on a PEG-BSA-coated cover glass [27]. Atto 532 also showed low aspecific interaction levels in labeling SNAP-tagged molecules in live cells [29] or with model lipid systems [26,102].

On the contrary, some dyes showed high levels of aspecific interactions in different works. An example is Atto 550, investigated using labeling of molecules exploiting either SNAP-tag [29] or ACP-based-tags [28], and using dialysis with model lipid bilayers [102]. Another case is Atto 647N, whose high spurious interactions were reported in labeling molecules in live cells via SNAP-tag [29], in labeling anti-EGFR affibody in live cells cultured on cover glass with or without PEG-BSA coating [27,85], in model lipid systems, such as lipid vesicles and supported lipid bilayers [26].

Interestingly, some dyes instead performed differently in different studies. Alexa 647 was considered a good choice for its low level of aspecific interactions when used for labeling proteins tagged with SNAP [29] or ACP-derived tags [28] in cells and also in a study on model lipid bilayers [102]. On the contrary, it interacted strongly with cationic lipids such as 1,2-dioleoyl-3-trimethylammonium-propane (DOTAP). Alexa 546 showed strong aspecific interactions when it labeled anti-EGFR affibody in live cells cultured on cover glass with or without PEG-BSA coating [27,85]. In contrast, it had aspecific binding with model lipid bilayers [102]. Finally, Alexa 555 turned out to be one of the dyes with the highest level of spurious interaction when used to label anti-EGFR affibody in live cells cultured on a PEG-BSA-coated cover glass [27], and at the same time, was one of the dyes with the lowest level of spurious interaction with model lipid bilayers [102].

Some of the mentioned works sought to determine if the physicochemical properties of the dyes could be used to predict the degree of aspecific binding. In a study by Zanetti-Domingues et al., aspecific binding effects showed a strong correlation with hydrophobicity and only a weak correlation with the net charge [27]. However, Hughes et al. observed a modest correlation with hydrophobicity and many outliers, so they stated that hydrophobicity, while important, was not a robust parameter for predicting dye behavior [102]. They also observed some impact of net charges, reactive groups and lipid compositions of the analyzed systems, but found no evident relationships. In concluding, they recommended a quantitative measure when choosing the probe. In another work, Bosch et al. noticed that the complex combined effect of local charges and polar and lipophilic groups of the dyes made it difficult to predict their aspecific binding level [29]. Some trends could be driven by the lipophilicity of a dye and others could be more influenced by localized electric charges (as also noted by Zhang et al. [26] and in general for protein adsorption by Quinn et al. [86]), and often a single property cannot be predictive. Bosch et al. did not even find a correlation between the fluorophore family and its aspecific interactions [29]. Additionally, Amodeo et al. detected high spurious binding levels of the hydrophilic dye Alexa 568, which instead resulted in low levels in other studies [102]. In that case, the aspecific adsorption of the dye was ascribed to its conjugation with CoA, required to label ACP-based tags [28]. Some dyes were investigated by Hughes et al. when conjugated with different reactive groups, and in some cases, they showed different aspecific levels: Alexa 532 and Atto 647 conjugated with succinimidyl ester or maleimide showed lower aspecific interactions in the first case, while for Alexa 647, no significant differences were observed for the two different conjugations [102].

This investigation of the literature highlights that the degree of aspecific interactions of the dyes is highly variable, its negative impact on single-molecule studies gets to be very severe, currently, and prediction of the phenomenon based on known properties is not reliable; therefore, an investigation of spurious behaviors of different dyes in the experiment of interest is necessary. We noticed that comparisons are usually performed by incubating the different considered dyes at the same concentration in the system of interest. However, this kind of evaluation does not take into account that different dyes usually have different efficiencies in labeling the molecule of interest [103] and would actually require different concentrations to get the same degree of labeling. For a more reliable comparison, concentrations of dyes corresponding to the same labeling efficiency should be used.

## 5. Fluorescent Probes for Single Molecule Microscopy

**Figure 2:**
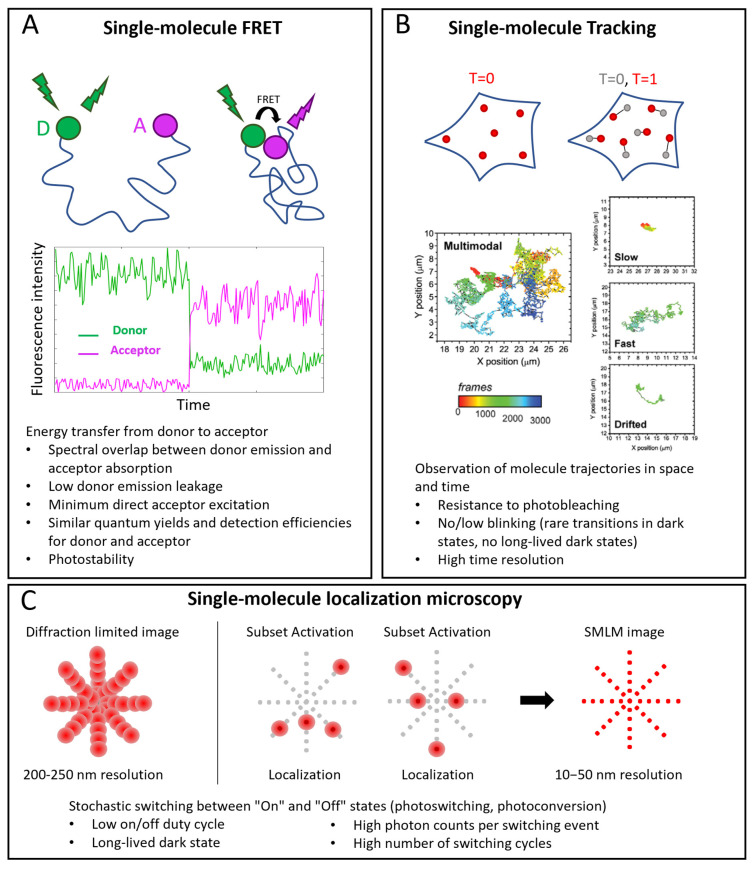
Single-molecule applications in microscopy. (**A**) smFRET. Top: example of conformational study: donor (D) and acceptor (A) probes label two sites of the same molecule. When the sites are distant, no FRET occurs: under D excitation, only its emission is detected; when the sites are near enough (typically a few nm), FRET occurs: when D is excited, emission of A is detected. Middle: example of corresponding single-molecule intensity traces. (**B**) Single-molecule tracking. Positions of labeled molecules are determined at different time points and their trajectories are reconstructed. Examples of tracks reproduced with permission from [10] (© 2013 The Company of Biologists Ltd.), where TrkA receptors were labeled with Quantum Dots. Multimodal tracks include periods of slow, fast and drifted motions. (**C**) Single-molecule localization microscopy (SMLM). Top, left: in conventional diffraction-limited images, resolution is around 200–250 nm, so that PSFs of close emitters overlap. Top, right: SMLM exploits activation of small subsets of probes at a time to have nonoverlapping point spread functions (PFSs), localize them individually and reconstruct images with 10–50 nm resolution. In the bottom part of each panel, specific probe requirements for each technique are highlighted.

The main fluorescent probes belong to three groups—fluorescent proteins, organic dyes, and nanoparticles—among which the most used are fluorescent semiconductor quantum dots (Qdots). In the following sections, we discuss and compare the features of the different probes, focusing on their advantages and weaknesses in single-molecule microscopy. Some requirements are common to all applications, such as high SNR, low aspecific interactions, specific, efficient, and controlled stoichiometry conjugation, availability of reactive forms and protocols for labeling, small size, and minimum perturbation on the system. Other requirements depend on the specific kind of application (Figure 2). Therefore, in the next section, we describe several single-molecule techniques with their specific requirements. Some features of the different probes, in particular considering their use in the discussed single-molecule techniques, are summarized in Table 3.

### 5.1. Single-Molecule Techniques

Three important classes of single-molecule techniques are single-molecule FRET (smFRET), single-molecule tracking (SMT, also called single-particle tracking (SPT)), and single-molecule localization microscopy (SMLM) (Figure 2).

FRET is a powerful approach for studying intramolecular and intermolecular interactions (Figure 2A). At the single-molecule level, it allows observation of rare states and characterization of detailed kinetics. FRET consists in an energy transfer from an excited fluorophore (the donor) to another one (the acceptor) when they are close enough (typically in the nanometer range). A spectral overlap between donor emission and acceptor absorption is necessary for an efficient transfer. Other requirements are: low cross talk between the channels (low donor emission leakage in the acceptor emission window and minimum direct acceptor excitation), high photostability (low photobleaching and intensity fluctuations), and, mostly in the single molecule case, similar quantum yields and detection efficiencies of donor and acceptor [106,107].

SMT consists in the reconstruction of the trajectories of single molecules (Figure 2B). An essential requirement is the ability to follow the molecules for enough time, so the probe must be resistant to photobleaching. Moreover, intensity fluctuations are not desirable because they cause interruptions in tracks. An accurate track reconstruction also requires a high time resolution: higher SNR allows shorter integration times per frame and a larger number of sample points for reconstructing tracks [104,108].

In SMLM, individual fluorescent molecules are localized with resolutions of the order of nanometers or tens of nanometers, starting from diffraction-limited images, thanks to the activation of small subsets of molecules per cycle [109,110] (Figure 2C). The fundamental requirement is a mechanism that ensures stochastic transitions between “Off” and “On” states, which must lead to sparse activations in order to have only one On-dye in a diffraction-limited area. This requires a low On/Off duty cycle (defined as the fraction of time a fluorophore spends in the On state) and long-lived dark states. Other requirements are high photon counts per switching event and generally a high number of switching cycles [32,111].

### 5.2. Fluorescent Proteins

The discovery of the green fluorescent protein (GFP) in the early 1960s revolutionized biological investigations, and in the last 30 years, it has seen extensive use [112,113]. Fluorescent proteins (FPs) from different species and several mutants have expanded the color palette of this kind of fluorescent reporter [114,115]. One of the advantages of FPs is their intrinsically specific labeling with controlled stoichiometry, thanks to the fact that their DNA sequence is genetically fused to the one of the molecule of interest. The only impact of aspecific labeling (see Section 4) could arise from the partial degradation of the chimeric protein. Moreover, the system can be used in many contexts, such as cells, tissue, yeasts, and bacteria, with better biocompatibility compared to the other kinds of probes. At the same time, there are some downsides. FPs are less bright and photostable than organic fluorophores or Qdots. They are also larger than organic fluorophores (even if smaller than Qdots), so they are more likely to introduce perturbation in some processes. Indeed, FPs are ~25 kD in size, while organic fluorophores have average sizes typically less than 1 kD [115]. Another critical point is that many FPs tend to oligomerize [116]. This tendency may depend on some experimental conditions, such as concentration, so an assessment of the phenomenon in the context of interest can be required to avoid artifacts [117,118,119,120].

In particular, for smFRET, the most common pair consists of a cyan-emitting variant (cyan fluorescent protein, CFP) and a yellow-emitting variant (yellow fluorescent protein, YFP) [121]. However, some limitations are associated with this FRET pair and multiple factors must be considered to allow quantitative measurements: overlap of donor and acceptor emissions, dependence of YFP properties on environmental conditions (e.g., pH), complex photokinetics such as reversible photobleaching or photoconversion of YFPs into CFP-like FPs, low brightness of CFP, and dimerization tendency [121,122,123]. Advanced versions of CFP and YFP have been developed to improve these factors, such as eCFP, Cerulean, mTurquoise, mCerulean3, mTFP1, Aquamarine for the first; EYFP, mVenus, mCitrine, sEYFP, YPet, for the second [121,122,124,125,126]. These reduced some of the mentioned weaknesses, although only partially. Green donors (GFP and enhanced versions eGFP, Clover, mNeonGreen) with red acceptors (such as mCherry, monomeric DsRed, RFP or mRuby) have been proposed and used as well [120,123,127]. This approach allows for lower induced autofluorescence, less phototoxicity, greater separation of spectra and resistance to environmental changes [122,124]. However, red FPs typically have low brightness, with a FRET emission that can be too weak even compared to the donor emission tail [124]. Recently, there have been efforts to develop new versions of red-emitting FPs to make up for the lack of bright and stable red fluorescent proteins, especially for FRET applications [128,129,130]. Therefore, even if several challenges still affect FP-based smFRET, the continuous development of new FPs gives hope that this approach will improve in the future.

Live-cell SMLM was enabled by the development of photoactivatable fluorescent proteins, which are still widely used in particular for photoactivated localization microscopy (PALM) [131,132,133,134]. Shroff et al. demonstrated PALM imaging in live cells using the photoconvertible fluorescent protein EosFP and they succeeded in observing nanoscale dynamics within individual adhesion complexes [132]. Employment of FPs was also favored by the ease of performing intracellular labeling in live cells, not possible with labeled antibodies or with many membrane-impermeable organic dyes and dye conjugates [135,136]. Several types of FPs suitable for SMLM have been engineered, including irreversible photoactivatable FPs (PA-FPs), photoconvertible or photoshiftable FPs (PS-FPs) and reversible PA-FPs (also called photoswitchable FPs) [109,137,138,139,140]. For example, the FP Dronpa (also its variants) is one of the best-known examples of PA-FPs. Its mechanism is based on a reversible cis−trans isomerization: intense irradiation at ∼488 nm converts Dronpa from the fluorescent cis-form to the non-fluorescent trans-form; illumination at ∼405 nm converts it back to the fluorescent form, a mechanism shared by other photochromic FPs derived from the GFP and its mutants [140,141,142,143]. Shroff et al. achieved two-color PALM simultaneously using EosFP and Dronpa to investigate spatial relationships between two different proteins in fixed cells. A summary of the photoswitching properties of selected FPs and an overview of their SMLM applications are available in [141].

FPs have been employed in SMT as well [144,145], even if their limited brightness and photostability usually lead researchers to prefer other kinds of probes for better SNR, time resolution and total observation time, especially for studies on molecules with faster diffusion. FPs are used in a specific type of SPT based on photoactivated localization microscopy, sptPALM, where the trajectories of a few photoactivated FPs are followed [134,146,147,148,149]. For their use in SMLM and SPT as well, there is a continuous effort to develop better-performing FPs allowing better signal-to-noise ratio and photostability, with special attention to new ones emitting in the red and infrared regions [150,151,152], especially for deep-tissue imaging.

### 5.3. Organic Dyes

Organic dyes form another class of fluorescent labels. They belong to different families, e.g., cyanines, oxazines, coumarins, rhodamines, BODIPYs [153,154]. They are produced by several companies, such as Molecular Probes (Alexa Fluor), ATTO-TEC, Abberior, and Dyomics, and are typically commercially available in diverse reactive forms (functionalized with NHS, maleimide, azide, carboxylic acid).

Organic dyes, in comparison to FPs, have higher brightness, less photobleaching, wider available spectral range and smaller size [155]. The main weaker aspect compared to FPs is that labeling is not genetically encoded, so it requires additional steps such as chemical coupling reactions; these can be laborious, require controls of labeling efficiency and lead to aspecific labeling and interactions [27,29,156]. Extensive washing is usually necessary to try to remove unbound dyes, but this must be mild for preserving cell adhesion and functioning, so it is usually not fully effective. Moreover, the time needed for washing makes time-dependent assays difficult. On the other hand, several complications arise when trying to treat surfaces to prevent aspecific dye adhesion (see Section 4). Labeling can be performed using antibodies chemically conjugated with fluorescent dyes (direct or indirect immunostaining [157]). This approach in live cells is only suitable for labeling molecules on the plasma membrane; intracellular labeling can only be applied to fixed (dead) cells. One of the main limitations of this labeling method arises from the relatively large size of antibodies (150 kDa, ~10 to 15 nm), which nullifies the advantage of organic dyes’ small size. To reduce the possible hindrance, it is possible to use smaller Fab (fragment antigen-binding) fragments (55 kDa, ~5 to 6 nm), or nanobodies (15 kDa, ~4 nm) [11,37,158]. Fab fragments have been used for single-molecule fluorescence microscopy in living cells, e.g., in labeling CD59 receptors with Alexa 647 [159] or CD36 receptors with Cy3 [160].

**Figure 3:**
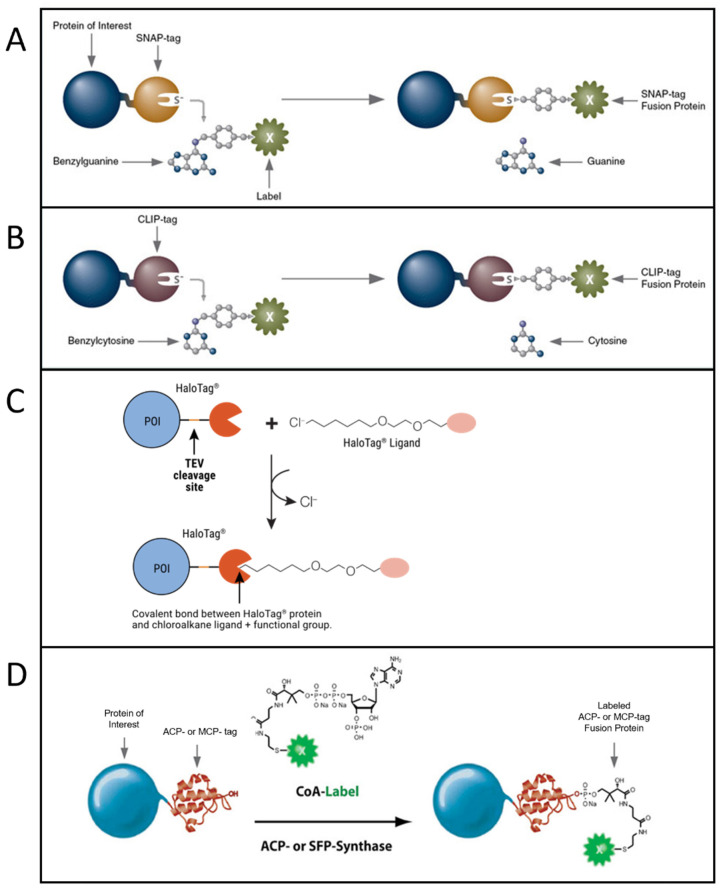
Most popular tag-based labeling strategies. (**A**) SNAP-tag. A benzylguanine—derivative of the label reacts with the self-labeling SNAP-tag fused to the protein of interest (image from https://www.addgene.org, accessed on 20 October 2022). (**B**) CLIP-tag. A benzylcytosine—derivative of the label reacts with the self-labeling CLIP-tag fused to the protein of interest (image from https://www.addgene.org, accessed on 20 October 2022). (**C**) Halo-tag. A chloroalkane—derivative of the label reacts with the self-labeling Halo-tag fused to the protein of interest (POI) (image from [179] used with permission from Promega Corporation), TEV: Tobacco Etch Virus. (**D**) ACP-tag. The enzyme ACP- (or SFP-) synthase catalyzes the attachment of a Coenzyme A (CoA)-label to the ACP- (or MCP-) tag fused to the protein of interest (image from https://www.addgene.org, accessed on 20 October 2022).

Another class of strategies to perform fluorescent labeling with organic dyes is based on the genetic insertion of a protein/peptide tag in a protein to be labeled. The tag is recognized and bound by specific chemical motifs, which are bound to or are part of the dyes. Requirements for this approach include small and non-perturbing tags; specific, efficient and fast labeling, even in the crowded cellular environment; and possibility of conjugating different kinds of probes in different cell compartments in vitro and in vivo [161]. Peptide tags have the advantage of being smaller than protein tags. A specific and strong conjugation between the dye and a short peptide tag is often achieved by exploiting enzymes that catalyze the covalent bond between them or by exploiting click chemistry. Extensive reviews about protein/peptide tags useful for fluorescent labeling can be found in [161,162,163]. The most used approaches, especially for single-molecule imaging, are based on SNAP-tag [164], CLIP-tag [165], HaloTag [166,167,168], and ACP and related tags [169,170] (Figure 3).

SNAP-tag is a 20 kDa (30-residue) self-labeling protein tag, engineered to react efficiently with benzylguanine (BG) derivatives, resulting in a covalent link (Figure 3A). Several popular organic dyes suitable for single-molecule applications are commercially available from New England Biolabs (Ipswich, MA, USA), as ready-to-use BG substrates, e.g., Alexa, Atto, or Dyomics dye derivatives. Additional ones can be prepared via a reaction of BG-NH2 (New England Biolabs) with N-hydroxysuccinimide ester (NHS) forms of the dyes (example of protocols is available in [171]), typically commercially available for dyes of practically all companies. SNAP-tag technology can be used for both extra- and intracellular labeling: in the first case, using non-cell-permeable substrates such as SNAP-Surface^®^ (New England Biolabs), for studying, e.g., membrane receptors [172,173]; in the second case, using cell-permeable substrates such as SNAP-Cell^®^ (New England Biolabs) or Janelia Fluor dyes, a quite recent family of cell-permeable fluorophores for single-molecule imaging [48] (protocols for the synthesis of SNAP-tag ligands of Janelia Fluor and subsequent labeling can be found in [47]).

SNAP-tag-based fluorescent labeling has been used in diverse single-molecule applications, such as single-molecule tracking in living cells (e.g., on G protein-coupled receptors, GPCRs [173] or epidermal growth factor receptors, EGFRs [174]), as well as smFRET [175] and SMLM (e.g., in live cell direct STORM, dSTORM, on core histone H2B proteins [176]). A comparison of different organic dyes to label SNAP-tagged proteins for multicolor single-molecule tracking in live cells was reported in [29].

CLIP-tag is another 20 kDa self-labeling protein tag engineered from SNAP as an orthogonal labeling system, accepting benzylcytosine (BC) derivatives as substrates (Figure 3B). By using the two tags, it is possible to obtain simultaneous and orthogonal two-color labeling with two different dyes. For example, in this way, Calebiro’s group performed a two-color single-particle tracking study on the interactions between a GPCR (labeled via SNAP-tag) and a G protein (labeled via CLIP-tag) [177]; Vafabakhsh et al. used smFRET to study the dimerization of metabotropic glutamate receptors (mGluRs), expressed simultaneously as SNAP-form and CLIP-form and labeled with FRET donor and acceptor dyes [178]. New England Biolabs provides cell-permeable dye substrates (CLIP-Cell™) suitable for intracellular labeling and non-cell-permeable dye substrates (CLIP-Surface™) for labeling on the cell surface.

HaloTag is a self-labeling protein tag that reacts specifically with chloroalkane (CA)-derivatized fluorophores (Figure 3C). Like SNAP and CLIP, it leads to specific and covalent linkage. It is larger than SNAP and CLIP, being 33 kDa and having 297 residues. A study reported a higher reaction rate for rhodamine substrates with HaloTag compared to SNAP and CLIP; however, HaloTag rate varied considerably with the substrates, while SNAP rate was less affected [103]. Complete protocols for HaloTag-based labeling (generating a HaloTag fusion protein, generating Halo fluorophore ligands starting from their NHS forms, labeling) are provided by Promega [180]. Protocols for single-molecule tracking inside the nucleus of living cells exploiting HaloTag labeling are available in the literature as well [181]. Some ready-to-use dyes for HaloTag labeling are available from Promega (including some for intracellular labeling, e.g., tetramethylrhodamine (TMR), Oregon Green, Janelia Dyes, and others for labeling on the cell membrane, e.g., Alexa 488 and 660). Examples of single-molecule applications with HaloTag labeling are single-molecule tracking on Halo-tagged-RING1B protein labeled with photoactivable Janelia Fluor 549 (PA-Halo-JF 549) [182] or on Halo-tagged-transcription factor SRF (serum response factor) labeled with TMR [183].

Another group of tags has been engineered from the acyl-carrier protein (ACP). These are enzyme-mediated labeling tags: phosphopantetheine transferase (PPTase) enzymes catalyze the covalent conjugation of a phosphopantetheine-dye to a serine residue in the tag (Figure 3D). The first developed tags were the peptidyl-carrier protein (PCP) and acyl-carrier protein (ACP, also with its mutant MCP) domains (80–120 residues, ~9 kDa in size and so smaller than SNAP, CLIP and Halo Tag) [170,184,185]. These tags were further optimized to obtain even shorter tags, such as the YBBR (11 residues), S6 (12 residues) and A1 (12 residues) [38,169,186]. In this group of tags, couples allowing orthogonal labeling with two different probes were identified, i.e., ACP-MCP or the shorter tags A1-S6. Orthogonality is achieved because two different enzymes, Sfp and AcpS, have to be used for the labeling [186]. Simultaneous orthogonal labeling of S6-tagged TrkA and A1-tagged p75 neurotrophic receptors was obtained [38].

Organic dyes in phosphopantetheine form can be obtained via conjugation with coenzyme A (CoA), obtainable from the maleimide reactive form of the dye reacting with CoA via a thiol-maleimide reaction. Protocols for production of PPTase enzymes AcpS and Sfp, synthesis and purification of CoA–dye conjugates, and expression and labeling of tagged proteins can be found in [187,188].

CoA-conjugated probes are not cell-permeable, so labeling of ACP-derived tags in living cells can be performed only on the cell membrane. This has been applied in single-molecule studies with small organic dyes on the cell membrane, e.g., in single-molecule tracking in living cells on S6-tagged p75NTR receptor [35], ACP-tagged TrkA receptor [10,189], ACP-tagged integrins [30], ACP-tagged D2R (a GPCR) [190], precursor and mature YBBR-tagged nerve growth factor (proNGF and NGF, followed also in endocytic vesicles) [191,192] and also in smFRET on S6- and A1-tagged HIV-1 Gag [193], on ACP-tagged CD59 [194], on YBBR and S6-tagged RNA polymerase II, TFIIF (transcription factor II F) and TFIIB (transcription factor II B) [195].

The different tags have also been combined to achieve multicolor and orthogonal labeling, going beyond the standard use of SNAP–CLIP and ACP–MCP or S6–A1 couples. Single dynein molecules were labeled simultaneously with Halo and YBBR tags [196] and with SNAP, Halo and YBBR tags for three-color imaging [197]; SNAP-β2-adrenergic receptor and HALO-transferrin receptor were orthogonally and simultaneously labeled [198].

An issue not fully understood is the effect of different tag systems on the optical and photophysical properties of a dye. Benke et al., considering the same dye and the same protein to be labeled, did not detect differences in dye performance when using different tags (ACP, Halo, SNAP) for SMT of stochastically active dyes [198]. However, Erdmann et al. considered silicon rhodamine (SiR) derivative dyes in STED microscopy and measured a higher brightness for Halo-tagged proteins compared to SNAP-tagged ones, while they did not detect significant differences in brightness between Halo and SNAP tags for other rhodamine-based dyes (TMR, JF549) [199]. The HaloTag-JF549 performed better for SMT experiments than the SNAP-tag- or CLIP-tag-JF549, showing higher photostability and lower aspecific binding [200].

The differences eventually observed between the different tags are dependent on both the labeled protein and the labeling dye [199]. These differences could stem from the different fluorophore environments generated by the tags [200], since it is well known that different dyes have very variable sensitivity to environmental conditions [199,201,202] or to different kinds of interactions between the probe and the tag [103].

**Figure 4:**
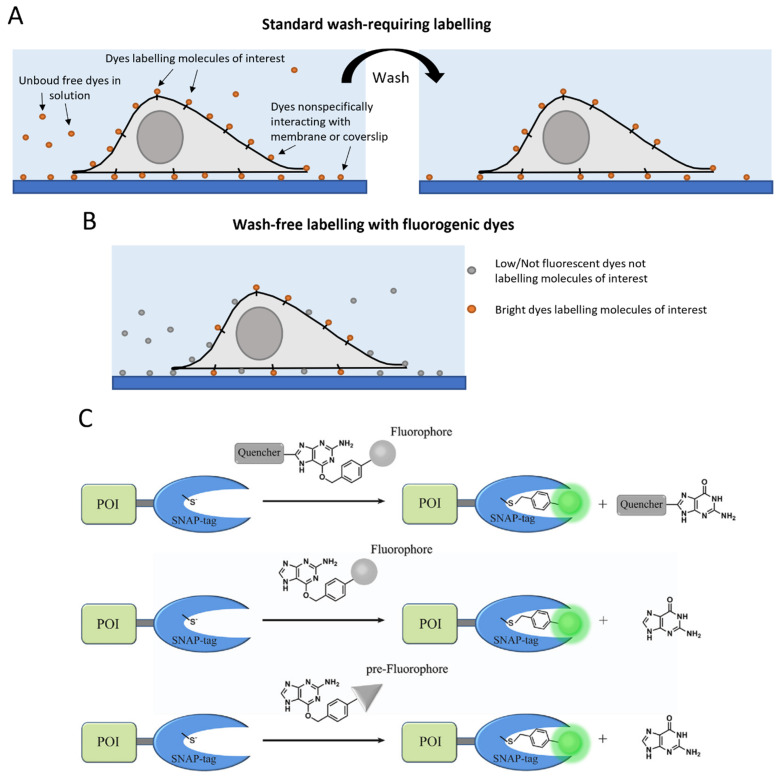
Aspecific interactions during labeling and improvement provided by fluorogenic dyes. (**A**) Labeling with conventional dyes. Left: the image shows a cell with some target molecules (small black lines) on its membrane. In the labeling reaction, some dyes (orange circles) label the molecules of interest, some remain free in solution, and some interact aspecifically with the cell membrane or the coverslip. Right: After washing, the free dyes in solution are removed; some aspecifically interacting dyes are removed but others remain. (**B**) Fluorogenic dyes allow low background, wash-free labeling. Dyes aspecifically labeling molecules of interest are dim or not fluorescent. (**C**) Example of mechanisms for obtaining fluorogenic dyes: fluorophore quenching with quencher released upon labeling (top); environmental sensitivity of the fluorophore (middle); in situ fluorophore generation (bottom). Image in panel C is reprinted from [210] with permission from Elsevier, 2017. POI: protein of interest.

In recent years, a series of studies proposed a very interesting solution to the problem of aspecifically interacting dyes (Figure 4A) through the development of fluorogenic dyes. These special dyes are able to activate (or very significantly increase) their fluorescence only upon labeling the molecule of interest, thanks to mechanisms such as activation by enzymes, environmental sensitivity, quenching (e.g., by FRET) with quencher released upon labeling, in situ fluorophore generation, and structural changes upon labeling [203,204,205,206] (Figure 4B,C). This strategy enables wash-free imaging with low background, which is particularly useful for single-molecule studies. In some cases, the dyes have been designed for a specific tag, in particular Halo or SNAP. Bachollet et al. reported the development of red and far-red fluorogenic dyes with fluorescence enhancement up to 156-fold upon conjugation with HaloTag [207]; Liu et al. designed a fluorogenic probe with 1000-fold fluorescence enhancement upon the same reaction [208]; a very recent study presented a palette of fluorogenic HaloTag labels with emissions spanning the green to the red range [209]. Other works focused on the development of fluorogenic probes for SNAP-tag [210], e.g., the solvatochromic dye Nile Red was exploited to develop a fluorogenic probe that fluoresces only after conjugation with SNAP-tagged membrane proteins [211]; Liu et al. exploited an environment-sensitive fluorophore to obtain a cell-permeable fluorogenic probe showing a 280-fold turn-on upon labeling SNAP-tags [212]; another fluorogenic probe was created on the basis of the Kaede red fluorescence protein chromophore, with a 90-fold enhancement in fluorescence intensity upon conjugation with SNAP [213]; Qiao et al. developed a fluorogenic probe for SNAP-tag with the fastest labeling rate among fluorogenic probes [214].

In other cases, the strategy adopted for the creation of fluorogenic dyes can be applied to more than one tag. A class of fluorogenic dyes, called MaP dyes, was developed by exploiting the equilibrium between a fluorescent zwitterion and a nonfluorescent, cell-permeable spirolactam, typical of rhodamine dyes: this strategy was used for both Halo-labeling and SNAP-labeling versions of the fluorogenic dyes, even at different wavelengths [215].

Organic dyes have been widely employed in single-molecule applications. Different couples of organic dyes have been used for smFRET, like the very popular Cy3/Cy5 or Atto 550/Atto 647N, but also Alexa 555/Alexa 647, Atto 532/Atto 647N, Atto 488/Atto 647N, Alexa 488/Alexa 594, Alexa 488/Alexa 555, Alexa 488/Atto 647N [25,58,107,216,217,218,219,220]. Förster radii of couples of Atto dyes can be found at [221], and of couples of Alexa dyes can be found at [222].

Even multicolor smFRET has been achieved with organic dyes. The first demonstration of three-color smFRET exploited one donor (Cy3) with two acceptors (Cy5 and Cy5.5), which labeled the three arms of a Holliday junction for simultaneously measuring distance changes between the donor and the two acceptors independently [223]. A weakness of this approach is caused by the spectral overlap of Cy5 and Cy5.5; therefore, better configuration was achieved using a Cy3/Cy5/Cy7 combination [224], even if Cy7 has a limited photostability. Four-color smFRET was employed to investigate two fully independent molecular interactions, by using Cy2 as an additional donor [225] or with a combination of the Atto dyes 488, 550, 594, 647N [226]. There are other applications of three-color smFRET in different in vitro systems, such as ribosomal assembly [227], ATP synthesis [228], intrinsically disordered proteins [229], activity and folding of 10–23 deoxyribozyme [230]. On the other hand, four-color smFRET studies are still very limited [226,231]. Multicolor smFRET is a promising area, although it still requires improvements in dye properties (like suitable spectra and photostability), labeling chemistry, instrumentation and data interpretation for easier applications in vitro and extensions into living cells.

Organic dyes were used in many more SMT experiments than FPs. They allowed tracking a variety of biomolecules on the cell membrane and in different cell compartments, in order to study several processes, among which an established example is diffusion and interactions of receptors [35,177]. Their current levels of brightness and photostability limit simultaneous multicolor studies or registration of very long trajectories (for these purposes, Qdots are more often exploited, as discussed in the following). Tracking duration is typically limited to about 10 s. However, there is a continuous effort to improve the photostability of organic dyes. It is known that their photodegradation can be caused by both oxygen-dependent and oxygen-independent processes and that the triplet state plays a fundamental role, although higher excited states can be involved as well. The exact mechanisms are not fully understood and depend on the specific conditions. Approaches for improving photostability are based on deoxygenation methods or on the use of additives in the medium such as antioxidants, triplet state quenchers, oxidizing and/or reducing agents [232]. A recent study performed a comparison of the photostability of organic dyes in SMT, searching for the best conditions using deoxygenation and a reducing-plus-oxidizing system (ROXS). The optimal solution was obtained with the dye SeTau 647, for which low oxygen concentrations (2%) plus ROXS allowed a sixfold prolongation of tracks, up to the order of minutes [30]. The other investigated dyes, in the optimal conditions for each one, showed lifetimes of 3–40 s.

Organic dyes are used in SMLM as well, in particular in stochastic optical reconstruction microscopy (STORM) [233]. The principle of STORM is similar to that of PALM, but in this case the photoswitching of fluorophores is random and requires special buffers, which are not suitable for living cells [32,135]. The most used formulations consist of deoxygenated solutions containing a thiol, where deoxygenation is achieved by enzymatic (e.g., via glucose oxidase and catalase, galactose oxidase, Oxyrase) or nonenzymatic (e.g., via methylene blue and a thiol) approaches; different types of thiols have been used (e.g., β-mercaptoethylamine, cysteine, glutathione) [141]. These buffers work for several but not all dyes, and the effects of the different buffer components on the photochemical mechanisms are not well understood yet. Different dyes can require different conditions for optimal photoswitching, either in buffer composition or additive concentrations [141]. Dempsey et al. found that the switching properties of 26 analyzed organic dyes varied with thiol type and concentration depending on the considered dye [32]. Atto 488 and Alexa 647 showed better (lower) duty cycles in the presence of mercaptoethylamine (MEA) compared to β-mercaptoethanol (βME), while DyLight 750 showed an opposite tendency. Atto 647N and Alexa 750 produced good quality STORM images in the presence of βME, while they were practically not usable with MEA. Low concentrations of MEA resulted in ameliorated switching properties, but high (≳100 mM) concentrations had a negative impact on some dyes [32].

Buffer formulations different from deoxygenate-thiol solutions have also been employed. They can be based on non-thiol additives, such as ascorbic acid and methyl viologen [234], on further addition of polyunsaturated hydrocarbon cyclooctatetraene (COT) to common buffers [235,236] or on the use of TCEP (tris(2-carboxyethyl)phosphine) [237]. Lists of imaging buffers for STORM can be found in [135,233] and some ready-to-use commercial formulations are also available for STORM on selected dyes [238,239]. However, buffers should be optimized for obtaining the best STORM conditions on the probe and the conditions of interest, especially when new experiments are implemented. Alexa Fluor 647 or Cy5 are typically recommended as first choices, being the most used and characterized dyes for STORM [109,141]. A summary of the photoswitching properties of selected organic fluorophores and an overview of their SMLM applications are available in the literature [141].

A recent field of research focuses on the development of spontaneous blinking dyes, which require no special buffers nor additives and not even high excitation power to perform SMLM; therefore, they can allow more flexible and cell-friendlier studies. A rhodamine-based spontaneously blinking fluorophore (SBF), named HMSiR, functions by exploiting the thermal equilibrium between a fluorescent open form and a nonfluorescent spirocyclic form [240]. HMSiR exists mainly in the nonfluorescent form and is converted stochastically into the fluorescent form emitting in the NIR region. The dye can be used in minimally invasive single-color SMLM in living cells, e.g., HMSiR was used in a light sheet microscope to obtain superresolution imaging of neurons in the intact brain of Drosophila melanogaster [241]. Uno et al. expanded the same principle to a green-emitting analogous dye named HEtetTFER [242]. In combination with HMSiR, HEtetTFER allowed two-color SMLM in fixed cells without additives or photoactivation.

Another principle for spontaneous blinking, developed by Morozumi et al., is based on the thermal equilibrium of intermolecular nucleophilic addition/dissociation of intracellular glutathione (GSH) on xanthene fluorophores, which show a fluorescent dissociated form and a nonfluorescent GSH-adduct form [243]. The authors developed blinking dyes emitting in the NIR and green range, SiP650 and CP550, with their HaloTag ligands form. They achieved two-color SMLM in living cells by combining either these two dyes or the NIR previously developed HMSiR with the green CP550.

Bewersdorf’s and Schepartz’s groups achieved live-cell two-color SMLM using HMSiR coupled with another new SBF, named Yale676sb, which emits in the near-IR and can be excited at the same wavelength as HMSiR with an adequate emission separation [244].

Some studies provided theoretical tools for accelerating the design of these recent SBFs [245,246]. These can guide the difficult tuning of the conditions for spontaneous blinking based on spirocyclization equilibria, reducing the number of derivatives needed to be synthesized and screened. Of note, some works even achieved the combination of fluorogenic and spontaneous-blinking properties on some dyes [247,248].

Another way to perform SMLM with organic dyes without special buffers is the method of point accumulation for imaging in nanoscale topography (PAINT) [249,250]. Instead of using photoswitching, this technique exploits stochastic and transient binding to the target of freely diffusing probes to visualize small subsets of molecules at a time. This allows more freedom in the dye choice. The most recent approach is DNA-PAINT, where fluorescently labeled DNA oligonucleotides diffuse freely in solution and can bind transiently to targets labeled with complementary DNA strands [251,252,253].

### 5.4. Quantum Dots and Other Nanoparticles

Quantum dots (QDs) have the great advantage of being brighter than organic dyes (and FPs), thanks to much higher extinction coefficients; moreover, they are not affected by photobleaching. The main limitation concerns their relatively large size. Functionalized QDs usually are 15–50 nm in size (diameter), bigger than organic dyes (<1 nm) and even than FPs (about 4 nm) [254,255].

**Figure 5:**
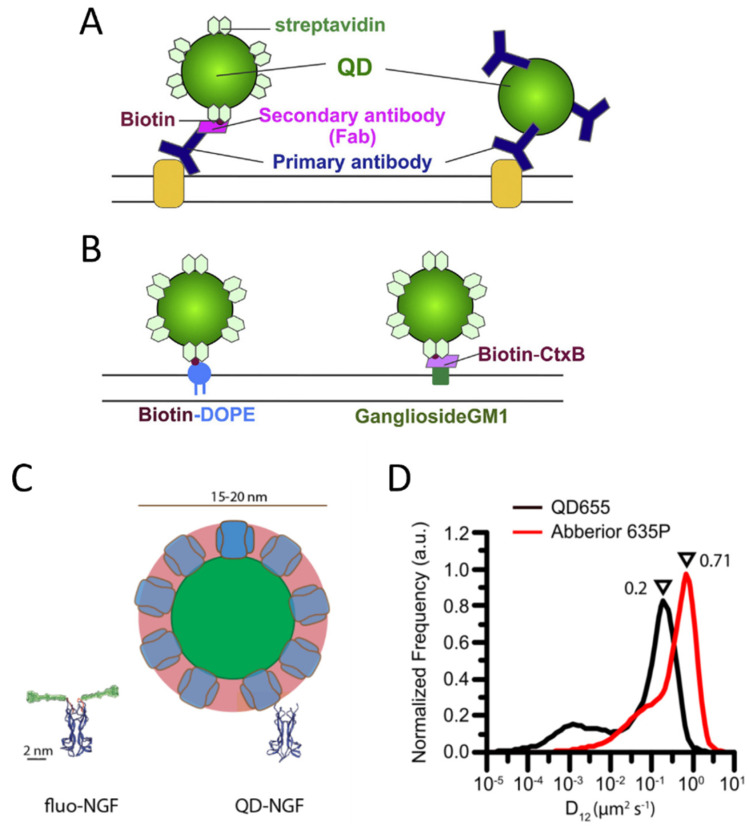
Labeling of membrane molecules with quantum dots. (**A**) Example of labeling based on antibodies. Left: a membrane molecule is recognized by a primary antibody, a biotinylated Fab fragment of the secondary antibody binds to the primary antibody, and streptavidin quantum dot (QD) binds to biotin. Right: QD is directly conjugated with the primary antibody. (**B**) Labeling of biotinylated molecules with streptavidin QDs. Labeling of a phospholipid (left) and a lipid raft component, the ganglioside GM1 recognized by a biotinylated cholera toxin B subunit (CtxB, right), are reported as examples. (**A**,**B**) are reprinted from [261] with permission (copyright 2018, Elsevier). (**C**,**D**) represent examples of hindrance caused by QD labeling. (**C**) Visual comparison between YBBR-tagged-NGF (nerve growth factor) labeled with organic dye and biotinylated NGF labeled with streptavidin QD (only backbone represented for NGF and tag, reprinted from [191]). (**D**) Labeling by QDs causes a slowing down of p75NTR receptors compared to labeling by smaller organic dyes, with a maximum in the reported measured diffusion coefficient distributions that decreases from 0.71 to 0.2 µm^2^/s (reprinted from [35]).

The most used methods for labeling with QDs exploit antibodies or the streptavidin-biotin interaction (Figure 5A,B). In the first case, the QD can be functionalized with a primary antibody for the molecule of interest, or with a secondary antibody; in the second case, the QD is functionalized with streptavidin, which stably binds biotin molecules: many biomolecules are already conjugated with biotin or can be biotinylated, e.g., thanks to peptidic tags [170,256]. QDs conjugated with streptavidin or with different kinds of antibodies are commercially available in ready-to-use formulations (e.g., by Thermofisher). SMT studies in live cells have been reported with both labeling strategies [257,258,259,260].

Different strategies for labeling with QDs were considered as well, which usually start from available reactive derivatives of QDs for custom conjugations, such as carboxyl- or amine-derivatized QDs. For example, the enzyme cutinase was inserted into the target molecule and its enzyme suicide substrate was conjugated with maleimide-activated QDs, in order to realize labeling of the cutinase molecule with QDs [262]. However, this approach still requires an entire enzyme and its substrate as a linkage between the molecule of interest and the QDs; therefore, it does not reduce the possible perturbations and steric hindrance effects of the relatively large streptavidin and antibodies molecules discussed above.

Some works tried to develop alternative strategies to overcome this issue and realize covalent labeling without using large proteins. Sunbul et al. started from commercial amino PEG-functionalized QDs to synthesize maleimide QDs, then, analogously to the ACP-based labeling described for organic dyes, they conjugated maleimide-QDs with coenzyme A so that they could label with QDs the protein tagged with the short tags S6, A1, PCP [263]. The bond between the protein and the QD consisted of the short and flexible phosphopantetheinyl (Ppant) linkage. However, there were some difficulties with respect to the case of organic dyes. Each QD presents more CoA molecules; therefore, multiple receptors can link to the same QD resulting in crosslinking. The authors observed that the density of CoA molecules on QDs depended on the CoA:QD ratio used in their conjugation reaction, but they could not measure the exact stoichiometry of CoA on the QD surface [263]. Moreover, they found that their density affected the efficiency of enzymatic labeling, and in some cases, attempting to have a small number of CoA on a QD to minimize crosslinking resulted in too-low labeling efficiency. Unknown molecule-to-probe stoichiometry and low and/or unknown labeling efficiency are problematic points, especially for quantitative single-molecule microscopy applications.

Behaviors similar to the one described above were also observed in approaches based on QDs functionalized with HaloTag ligands [264]. In those cases, streptavidin QDs were conjugated with biotin Halo ligands (at different QDs:ligands ratios) for a subsequent label of HaloTag proteins. Ligand density on QDs was variable and affected labeling efficiency in this case as well: only high ligand densities on QDs ensured acceptable labeling efficiency.

Therefore, alternative strategies for covalent labeling with QDs still need improvement and, in general, are not usually employed. Conjugations via antibodies or streptavidin QDs interacting with biotinylated molecules remain the most common approaches, in particular for labeling in cells.

Since extracellular targets are accessible to QD labeling, many SMT studies exist on various membrane molecules, such as receptors [259,265], ion channels [266,267] and lipids [268,269], resulting in longer tracks in comparison to organic dyes [255]. On the other side, the internalization of QDs and QD conjugates into the cytoplasm of living cells for intracellular imaging is challenging. Several methods have been tested, e.g., electroporation, microinjection, liposome fusion, and cell-penetrating peptides, but these approaches often cause QD aggregation and limited stability within the cytoplasm [270,271]. Some special alternatives to internalize QDs as single particles have been developed: Courty et al. employed internalization of QDs-kinesin based on the osmotic lysis of pinocytic vesicles to demonstrate intracellular SMT [272]. Yoo et al. exploited lipid-based transfection of QDs conjugated with phalloidin, anti-tubulin antibody and kinesin to perform intracellular SMT [273].

An intermittent behavior for QD emission (blinking) has often been reported [274], which could cause interruptions in track reconstruction. However, several strategies have been developed to reduce QD blinking, so the phenomenon has been improved over the years [275,276]. Moreover, tracking algorithms can afford the problem of temporary missing spots: especially in conditions of high SNR, as in the case of QDs, they allow for obtaining reliable tracking solutions [49,277].

**Figure 6:**
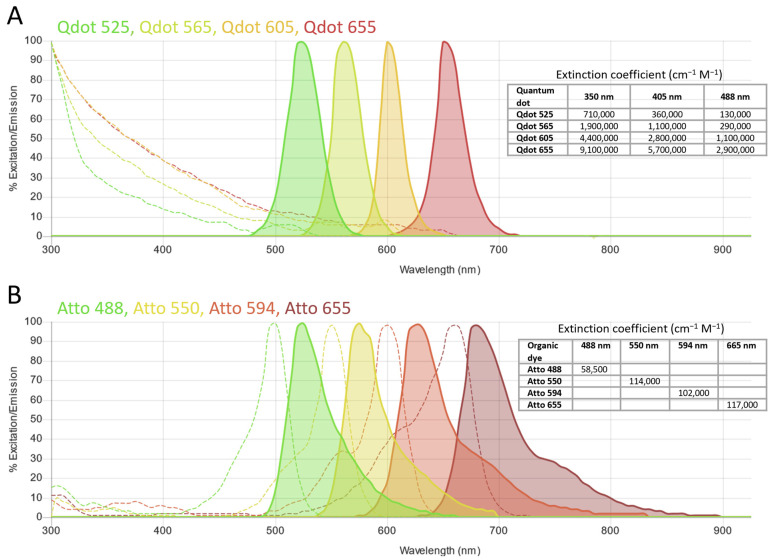
Comparison of spectra of quantum dots and organic dyes for multicolor studies. (**A**) Spectra of four distinct quantum dots (Qdot). Excitation spectra (dashed lines) are broad and with similar shapes. All the Qdots can be effectively excited using a single wavelength. Emission spectra (solid lines) are narrow, symmetric and quite well separated. (**B**) Spectra of four distinct organic dyes. Multicolor studies require multiple excitation wavelengths, one for each dye. The typical red tail is visible in the emission spectra, causing a greater overlap than for Qdot spectra. Spectra were produced with FluoroFinder Spectra Viewer [46]. *Y*-axis reports excitation (or emission) percentage of the excitation (or emission) spectra with respect to their maxima. Tables with extinction coefficients are added by us using data obtained from [282,283].

QDs were particularly preferred over organic dyes for multicolor SMT studies because they allow for easier implementation of the microscopy setup. Indeed, QDs have excitation spectra that increase towards the UV, such that probes emitting in different colors can be excited with one single wavelength; moreover, contrary to organic dyes, QDs have relatively narrow and symmetric emission spectra, without a tail at longer wavelengths, so many different channels can be better separated without spectral overlap [260,278,279,280,281] (Figure 6).

However, some studies highlighted perturbations of biomolecule behavior caused by QD labeling, e.g., QD labeling based on biotin–streptavidin conjugation caused a decreased diffusion coefficient for p75NTR receptors compared to labeling with small organic dyes on S6-tag, as observed with SMT on the membrane of living cells [35] (Figure 5D). Hindrance of receptor mobility has also been observed on B cell receptors, on which labeling with Fab–biotin–streptavidin QDs caused alteration in diffusion features in comparison to labeling with Fab-organic dyes [284]. A study employing single-molecule imaging and tracking of YBBR-tagged neurotrophins labeled with a small organic dye revealed that their signaling endosomes contain clusters of NGF made of 2–8 dimers [191], while a previous study with streptavidin-QD labeling of biotinylated NGF detected a single NGF dimer per vesicle [285]. The discrepancy has been explained on the basis of the different sizes and steric hindrance of the labeling (Figure 5C): QD labeling introduces a volume up to 70 times larger than NGF; using smaller organic dyes might allow accommodating a physiologically higher number of clustered NGF molecules in the same vesicle [191]. In another work, SMT on (GFP- and bungarotoxin binding site-tagged) GluR2 AMPA receptor subunits was performed comparing three different labeling approaches, i.e., QDs coupled to anti-GFP antibody (antibody size, 150 kDa), Cy5 coupled to anti-GFP antibody and Cy5 coupled to bungarotoxin (BTx, 8 kDa). A set of highly diffusing receptors was only detectable with the last smallest labeling complex (based on organic dyes and involving neither QDs nor antibodies), and this allowed the obtaining of a broader range of diffusion coefficients compared to the other labeling approaches [286]. The impact of more cumbersome labeling may depend on the considered system and the measurements of interest. Abraham et al. demonstrated that, even if QDs labeling caused a general slowdown, it allowed detecting large differences in receptor mobility, such as changes caused by destruction of the actin cytoskeleton, on par with organic dyes [284]. The impact of QD-labeling complexes is likely more evident on small, fast-diffusing and flexible molecules [35], while others can be less or not significantly influenced. Indeed, as mentioned before, several SMT studies have been performed using QD labeling and obtained relevant results [259,265,266,267,268].

The impact of labeling with Qdots can be compared to the one of less invasive labeling, e.g., based on organic dyes, considering the distribution of diffusion coefficients: this showed to be sensitive to cumbersome probes, is typically the first and most basic measurement from an SMT experiment, and its determination provides reasonable results also with dyes in spite of the shorter obtained trajectories or the higher localization uncertainty (caused by their lower brightness).

The application of QDs in FRET and smFRET is not as widespread as for organic dyes or FPs [107,254,287,288]. The typical configuration involving QDs is based on a QD as donor and a different kind of probe (often an organic dye) as acceptor [289,290]. Indeed, the employment of QDs as donors benefits from their high extinction coefficient for efficient donor excitation, their broad excitation spectra for efficient excitation/emission light separation, and their narrow and symmetric emission for cross-talk minimization. On the contrary, their use as acceptors is very challenging: their high extinction coefficient and broad excitation spectrum make it nearly impossible to avoid direct acceptor excitation upon excitation of the donor fluorophore. A recently introduced possibility consists in the exploitation of a different class of fluorophores as donors, the very long-lifetime lanthanide chelates: these allow suppression of the signal arising from direct QDs acceptor excitation by using time-gated data acquisition [291,292].

Reported examples of smFRET studies with QDs as donors and organic dyes as acceptors are: (i) smFRET between QD585 and Cy5 on Holliday junctions [293]; (ii) an investigation of the kinetics of ligand–enzyme binding at the single-molecule level using QD520-ATPase (donor-labeled enzyme) and Cy3-ATP (acceptor-labeled ligand) [294]; (iii) a study using Alexa 594 acceptor-labeled double-stranded DNA directly coupled to the QD553 donor through a C6 thiol linker [295]; (iv) a study in live cells of EGFR dimerization also using smFRET with QD605 donor-labeled EGF and Cy5 acceptor-labeled EGFR to observe the association between activated and inactivated receptors [296].

However, several challenges still limit the use of QDs in smFRET, mainly their large size (negatively affecting FRET efficiency and potentially causing perturbations), often the lack of a monovalent conjugation scheme, and a complex delivery inside cells for intracellular studies [254,297,298,299].

QDs are less used than FPs or organic dyes in SMLM [109]. QDs typically exhibit intrinsic blinking; however, exploiting this property for STORM has proven difficult. Indeed, QDs show a small Off/On time ratio, which makes it hard to create the desired large population of Off-probes. As a result, multiple emitters overlap in a diffraction-limited volume, limiting the ability to localize single contributions [71,300]. Moreover, QDs cannot be photoactivatable/switchable [300]. Therefore, alternative strategies have been developed for their use in SMLM. The most popular one exploits a blue-shifting property observed for QDs: under steady illumination, their emission continuously shifts toward shorter wavelengths and the phenomenon is spatially and temporally random in a sample. As such, by selecting a detection window at shorter wavelengths than the QD initial emission, it is possible to operate a sequential selection of subsets of probes (i.e., those emitting in that range) according to the main principle of SMLM [301]. The effective “Off-time” is increased with this strategy because it includes the real Off-time and the time the emission is outside the spectral detection window. The principle has even been applied in a two-color implementation [302]. The technique benefits from the high brightness of QDs, which allows a better localization accuracy than other SMLM methods (like PALM, STORM) that use FPs or organic dyes. Another approach for using QDs in SMLM is an analytical variant named quantum dot blinking with three-dimensional (3D) imaging (QDB3), which exploits a subtraction of adjacent frames in a movie to identify and localize single-blinking QDs [303]. Indeed, even if multiple emitters can be in the On-state in the same diffraction-limited spot, it is not likely that multiple of them blink simultaneously, so a subtraction with an adjacent frame allows resolution of a single blinking QD. In conclusion, even if the use of QDs in SMLM is not very common yet with respect to the other probes, recent and future developments in their design and in experimental and analytical techniques could make the exploitation of their favorable properties of higher brightness and photobleaching resistance more popular also in this application.

QDs are the most used inorganic nanoparticles in single-molecule microscopy. Of note, other kinds of nanoparticles have been used or are being developed, especially in SMT. These include upconversion nanoparticles (UCNPs), polymer dots (PDots), fluorescent nanodiamonds (FNDs). These are even brighter and more photostable than QDs. Tracking time ranges from tens of minutes up to hours with localization accuracies of a few nm [304]. A recent study exploited UCNPs to perform single-particle tracking on molecular motors in live cells, achieving 2.4 nm localization accuracy with 2 ms time resolution [305]. However, several challenges still limit the use of these powerful probes, mainly due to large sizes (UCNPs have sizes of 40–50 nm) and complex surface chemistries. As discussed for QDs, these features make it hard to obtain efficient and specific labeling or intracellular delivery and may induce perturbation on some labeled molecules, and these problems are even more severe than in the case of QDs [304].

## 6. Discussion

We reviewed the different aspects to consider probe choice in single-molecule fluorescent microscopy, with the final purpose of maximizing the specific signal while minimizing “noise” in a broad sense. The latter includes background, aspecific labeling and interactions, bleaching, interference with the biological system. We addressed these different contributions, discussing the present literature and suggesting avenues for further improvement.

We explained that an attempt to maximize SNR based on dye brightness alone is not a sufficient approach for single-molecule experiments, and showed other considerations needed to complement this evaluation. Efficient selection of dyes must include knowledge of their complete spectra alongside the spectra of efficiency of detection (due to transmission spectra of microscopy components and yield of the detectors). For this purpose, we suggest an optimal Spectra Viewer [45] with a comprehensive list of fluorescent probes, including the last developed or identified as optimal candidates for single-molecule imaging (e.g., Janelia and SeTau dyes) and with additional features (like detector quantum efficiency) compared to other online Viewer tools.

Importantly, we highlighted how the maximization of SNR requires knowledge of the (auto)fluorescence background associated with the experiment. We stress this point because there are too few investigations about this and improvements in this issue can push further the current potential of some single-molecule approaches. We discussed above all two important sources of background, i.e., biological samples and optical glasses, which require more extensive characterizations and are even more important to consider in simultaneous multiple-color applications. In conclusion, simply employing a generally adopted choice, without examining the background behavior in the specific sample and microscope of interest, can make an experiment infeasible.

We discussed another important factor to consider in dye choice, i.e., the level of aspecific interactions with substrates and lipid bilayers. We explained the effects of these phenomena on single molecule studies and the difficulties in acting a posteriori (after dye choice) by surface passivation. The literature reports some trends of spurious interaction levels with some dye properties, especially hydrophobicity. However, there are several involved factors and exceptions and a reliable prediction based on structures and properties of the dye is currently not possible. Therefore, the best approach should be to screen dyes identified as good candidates for other characteristics, such as SNR, in the experiment of interest. We suggested that comparisons of aspecific interactions should be best performed not by considering the same fixed concentration for all the investigated dyes, as typically done, but by considering for each one the concentration resulting in the desired labeling efficiency, which is typically different for different dyes.

We also discussed the choice of the fluorescent probe kind for different single-molecule fluorescence applications, by thoroughly examining the three typical groups of fluorophores: fluorescent proteins, organic dyes and quantum dots. The requirements for a fluorescent reporter, especially in these applications, include labeling specificity and efficiency, controlled stoichiometry, biocompatibility, no perturbation, high brightness and high photostability. Each kind of probe has its strengths and weaknesses. Fluorescent proteins benefit from specific and efficient labeling in live cells with controlled stoichiometry and high biocompatibility, thanks to their genetic encoding, but suffer from limited brightness and photostability. Organic dyes have better brightness and photostability; furthermore, they are smaller and therefore they minimize interferences with molecular functions. Established protocols are available for biomolecule labeling with organic dyes, with characterized conditions for achieving high efficiencies. Labeling specificity is typically affected by aspecific adsorption during the coupling reactions. A promising path relies on fluorogenic dyes, whose fluorescence is activated or increased only upon labeling the molecule of interest, even if these dyes still need improvement to become of common usage. QDs have even better brightness and resistance to bleaching compared to organic dyes. Their typical labeling strategies involve mediation by large molecules, such as antibodies or streptavidin; for this reason, together with their relatively high starting size, functionalized QDs reach much larger dimensions than the other probes. Therefore, an impact on the phenomenon under investigation is more probable.

The choice of the kind of label is crucially connected with the specific application. We discussed the employment of the different probes in different single-molecule areas, such as single-molecule FRET (smFRET), single-molecule tracking (SMT), and single-molecule localization microscopy (SMLM). Despite their limited properties in terms of brightness and photobleaching, which limit their use in SMT, FPs played a central role in SMLM, in particular in PALM, thanks to their photoactivatable mutants. Organic dyes have been used in diverse SMT applications to investigate a variety of biological processes; their main limitation concerns multicolor SMT or the recording of very long trajectories. In SMLM, organic dyes have been used in STORM, albeit with the requirement of special buffers to induce photoswitching, and in PAINT without the need for these special buffers. In smFRET, they are commonly used and even allowed three- or four-color configurations.

QDs are particularly powerful in recording long trajectories in SMT and have even allowed extensions to multicolor studies, thanks to their spectral characteristics. However, effects of molecular dynamics alteration have been observed in some SMT studies upon labeling with QDs in comparison to small organic dyes, likely due to the large size of QDs.

In conclusion, choosing a fluorescent probe for fluorescent microscopy is not a straightforward task, especially in the single-molecule limit. It requires a thorough investigation of different aspects. A suboptimal choice can drastically reduce the amount and accuracy of obtainable information, with more severe impact than in other less advanced microscopy applications. This review can help focus on the key points to consider, both in the implementation of a single-molecule experiment and in the development of new fluorescent probes [54].

## Figures and Tables

**Table 1:** Examples of published comparisons of some fluorescent probes in selected systems and applications; the investigated properties and the individuated best dyes are reported. (sm)FRET: (single molecule) Förster resonance energy transfer; SMT: single-molecule tracking; STORM: stochastic optical reconstruction microscopy; SMLM: single-molecule localization microscopy.

Considered System	Application	Analyzed Properties	Best Dyes	Ref.
Freely diffusing double-stranded DNA molecules	smFRET	Photostability, brightness, fluorescence lifetime and FRET performance	Alexa 488 and Atto 647N	[25]
Dyes on synthetic lipid membranes	Single-molecule studies	Aspecific interactions	Alexa 488, Atto 488	[26]
EGFR labeled via anti-EGRF affibody in live cells	SMT	Aspecific interactions	Alexa 488, CF640R	[27]
TrkA receptor labeled via ACP-derived tag in live cells	SMT	Aspecific interactions, mean fluorescence intensity and photostability	Abberior STAR 635p	[28]
EGFR labeled via SNAP tag in live cells	SMT	Aspecific interactions and photostability	Dy 549, CF 640	[29]
Integrins labeled via ACP-tag in live cells	SMT	Photostability	SeTau 647	[30]
Immunolabeled microtubules in fixed cells	STORM	Image quality	CF 555, CF 568, CF 647, Dy 547, Alexa 647	[31]
Immunolabeled microtubules in fixed cells	3-color STORM	Image quality	CF 568, CF 647, CF 680	[31]
Labeled antibodies adsorbed to coverslip; immunolabeled microtubules and clathrin-coated pits	STORM	Photon numbers, duty cycles, survival fractions and switching cycles	Alexa 647, Dy 654, Cy5	[32]
Labeled antibodies adsorbed to coverslip; immunolabeled microtubules and clathrin-coated pits	4-color STORM	Photon numbers, duty cycles, survival fractions and switching cycles	Atto 488, Cy3B, Alexa 647 and DyLight 750	[32]
Immunolabeled microtubules in fixed cells	SMLM	Image quality	Alexa 647, Cy5, Atto 488	[33]

**Table 2:** Published comparisons of dyes aspecific interactions. AL: Alexa, AT: Atto, Abb.: Abberior. SE: succinimidyl esters; HY: hydrazide; M: maleimide.

Considered System	Measurement	Worst Dyes, to Be Excluded from Single-Molecule Imaging	Best Dyes for Low Aspecific Interactions	Ref.
Labeling with anti-EGFR affibody-dye on cells grown on PEG-BSA nanogel surfaces	Diffusion coefficient in single-particle tracking	AL 546, AT 647N, AL 555, Cy3, CF 568, AT 565	AL 488, CF 640R, TMR	[27]
Interactions of dyes with lipid vesicles and supported lipid bilayers (diverse kinds of lipids)	Partition coefficient into the lipid membrane via fluorescence correlation spectroscopy and visualization of adsorbed dyes in TIRF	AT 647N, Cy5, AT 594	AT 488, AL 488, AT 532, Fluorescein	[26]
Labeling of receptors tagged with ACP-derived tags in cells	Incubation of not transfected cells with CoA dyes	AT 550, AL 568, AT 633	AL 488, AT 488, Abb. 488, Abb. 635p, AL 647	[28]
Labeling of SNAP-tagged proteins in cells	Incubation of non-transfected cells with BG-dyes	AT 550, AT 565, AT 620, AT 633, AT 647N, Dy 630, Dy 651, Abb. 635	AL 647, AT 532, Dy 634, Cf 633, Dy 649, Dy 648, Dy 549, Cf 640	[29]
Dye interaction with EggPC unilamellar vesicles	Dialysis on vesicle–dye mixtures	BODIPY-TMR M, AT 550 M, Cy 3 SE, AL 633 M, AT 647 M, sulforhodamine B, Texas Red M	AL 488 SE, AT 488 SE, AL 532 SE, AT 532 SE, AL 555 M, AL 568 HY, AL 647 SE, AL 647 M, Chromeo 488 SE, OG 488 SE, OG 514 SE	[102]
Labeling with anti-EGFR affibody-dye	Diffusion coefficients in cells	At 647 N, AL 546	AL 488	[85]

**Table 3:** Comparisons of some features for the three kinds of probes (fluorescent proteins (FPs), organic dyes (ODs) and quantum dots (QDs)) for single-molecule applications. Typical values are reported. Brightness for QDs is reported for visible excitation (405–488 nm). Sm-FRET: single-molecule Förster resonance energy transfer; SMT: single-molecule tracking; STORM: stochastic optical reconstruction microscopy; SMLM: single-molecule localization microscopy; D: donor; A: acceptor.

	FPs	ODs	Qdots
Brightness	10^3^–10^4^ M^−1^ cm^−1^	10^4^–10^5^ M^−1^ cm^−1^	10^5^–10^5^ M^−1^ cm^−1^
Photostability	Low	Medium	High
Size	4 nm	<1 nm	15 nm (up to 50 nm with functionalization)
Conjugation	Genetically encoded: specific, stoichiometry-controlled, easy also intracellularly.	Chemical coupling. Several protocols available for specific, efficient and stoichiometry-controlled labeling. Drawback: aspecific adsorption (resolved by fluorogenic dyes).	Chemical coupling typically requiring functionalization with large molecules. Challenges: intracellular delivery, control of stoichiometry and efficiency, steric hindrance.
Sm-FRET	Use of CFP-YFP couple and derivatives; research for improvement (bright red FPs) to overcome limitations.	Several available D-A couples, even multicolor FRET achieved.	Limited applications, used as donors with OD acceptors.
SMT	Limited applications due to poor SNR and photostability.	Many applications, not invasive. Drawbacks: short tracks, difficult multicolor. Typically: ~10 s of observation at 30–50 Hz with localization precision of 20–30 nm [30,35].	Longer tracks, easier multicolor. Drawbacks: possible perturbations on the labeled molecule, blinking. Typically: up to minutes of observation at 50–200 Hz with localization precision of about 10 nm [10,104].
SMLM	PALM with cell-friendly media. Localization precision: 10–50 nm [105].	STORM (using a photoswitching buffer); PAINT, DNA-PAINT. Localization precision: 20–30 nm [31,32].	Limited applications, specialized methods under development.

## Data Availability

Not applicable.
